# The COVID Catastrophe: What’s Gone Wrong and How To Stop It Happening Again, 2nd Edition

**DOI:** 10.3201/eid2711.211257

**Published:** 2021-11

**Authors:** Dongzhe Hong, Xin Yin, Lizheng Shi

**Affiliations:** Tulane University School of Public Health and Tropical Medicine, New Orleans, Louisiana, USA (D. Hong, L. Shi);; Pennsylvania State College of Medicine, Hershey, Pennsylvania, USA (X. Yin)

**Keywords:** coronavirus disease, COVID-19, severe acute respiratory syndrome coronavirus 2, SARS-CoV-2, coronaviruses, viruses, respiratory infections, catastrophe, zoonoses

After half of the adult population in the United States has been fully vaccinated against coronavirus disease (COVID-19) to date (*1*), we seem to finally see the light at the end of the tunnel, after a 1.5-year battle with the most unprecedented public health crisis in modern history. We are reckoning with devastating mid-pandemic chaos caused by avoidable mistakes that have added to adverse outcomes.

In this second edition of *The Covid-19 Catastrophe: What’s Gone Wrong and How to Stop It Happening Again*, Richard Horton, a medical expert and editor-in-chief of Lancet, leads us to revisit the COVID-19 crisis with his acumen, sharp arguments, and strong conscientiousness (Figure). Published in January 2021, this newer edition features updated epidemiologic numbers on COVID-19 and provides better data that account for discoveries and perspectives in the second half of 2020.

**Figure:**
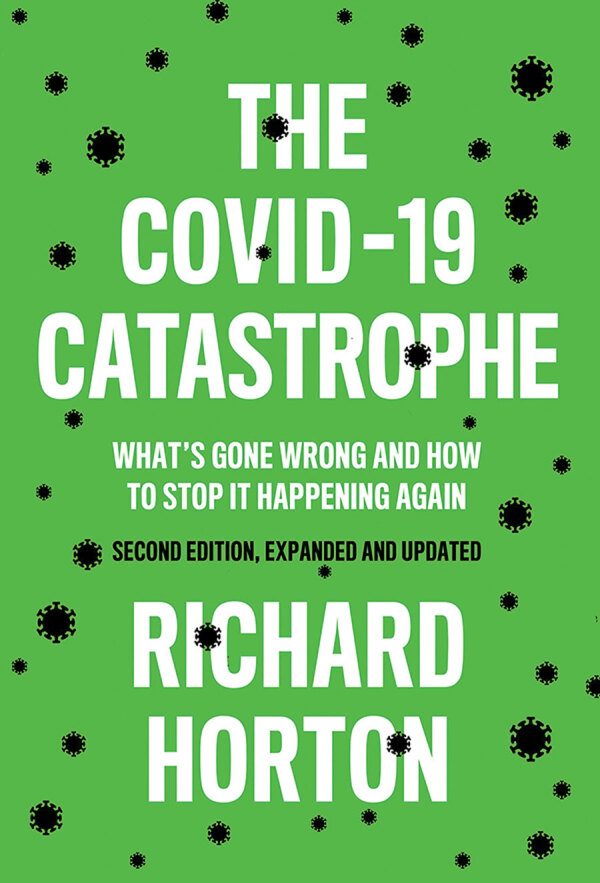
The COVID Catastrophe: What’s Gone Wrong and How To Stop It Happening Again, 2nd Edition

Before the main body, the author added a comprehensive introduction to explain the biology of the COVID-19 coronavirus and challenges of vaccine development and distribution. It also summarizes major lessons learned in response to COVID-19. Horton addresses awareness of the “terrible human cost” caused by lockdowns and potential long-term consequences that could afflict COVID-19 survivors.

Chapter 1 describes the origin of the COVID-19 pandemic by reviewing spread of the pandemic and responses from each country and the World Health Organization with a clear timeline. In Chapter 2, Horton engages the reader by asking, “Why were we not prepared?” A critical reason that most countries were unprepared was an underestimation of the danger of the coronavirus by political leaders and the general public, despite lessons learned from outbreaks of Ebola and Zika.

Chapters 3–5 further discuss the “disturbing twists” that might explain why countries were unprepared for COVID-19. Chapter 3 praises the efforts of frontline health workers and scientific groups in providing dependable knowledge regarding COVID-19 and criticizes indecisive policymaking in handling the emergency. In Chapter 4, Horton analyses responses of various countries and identifies elements needed to have a robust and resilient health system capable of responding to such a pandemic. Chapter 5 details the failures of government responses to COVID-19 and discusses how political misinformation played a role in the failure.

Chapter 6 offers a thought-provoking philosophy that emerging risks and problems we encounter today might result from our own developments (*2*). This chapter and the epilogue conclude with Horton’s opinions on the impact of COVID-19 on our future, and strategies that help prepare us for the next pandemic. It is indispensable for the World Health Organization to strengthen its global coordinating role by mobilizing resources and establishing an accountability mechanism.

The book is replete with straightforward facts and honest, bold, and sometmes furious arguments that show how COVID-19 is much more than merely a health crisis. “It is a crisis about life itself,” Horton writes. Although Horton makes some repetitive points throughout the book, we found it informative and a compelling reflection on the COVID-19 pandemic. It is a fascinating read for health professionals and nonhealth professionals who wish to understand the full scale of the cataclysmic pandemic we are currently experiencing.
